# Does completion of sputum smear monitoring have an effect on treatment success and cure rate among adult tuberculosis patients in rural Eastern Uganda? A propensity score-matched analysis

**DOI:** 10.1371/journal.pone.0226919

**Published:** 2019-12-26

**Authors:** Jonathan Izudi, Imelda K. Tamwesigire, Francis Bajunirwe

**Affiliations:** Department of Community Health, Faculty of Medicine, Mbarara University of Science and Technology, Mbarara, Uganda; The University of Georgia, UNITED STATES

## Abstract

**Background:**

Tuberculosis is a global public health problem. Bacteriologically confirmed pulmonary tuberculosis (BC-PTB) patients require three sputum smear monitoring (SSM) tests to establish cure or treatment success, but few studies have assessed the relationship. We evaluated the effect of completing SSM on treatment success rate (TSR) among adult BC-PTB patients in rural eastern Uganda.

**Methods:**

We conducted a propensity score-matched (PSM) analysis of a retrospective observational cohort data. Participants who completed SSM were matched to those who had not, through nearest neighbor 1:1 caliper matching. Balance of baseline characteristics between the groups was compared before and after PSM using standardized mean differences. Logistic regression analysis was performed in matched and unmatched samples, reported as odds ratio (OR) with 95% confidence intervals (CI). Robustness of the results to hidden bias was checked through sensitivity analysis. The primary outcome was TSR (treatment completion or cure), while the secondary was cure rate, measured as an individual outcome.

**Results:**

Before PSM, 591 (72.3%) of the 817 participants had incomplete SSM, with statistically significant differences in baseline covariates between completers and non-completers. After PSM, there were 185 participants in either group, balanced on baseline covariates. Before PSM, SSM completion was not associated with TSR, with unadjusted (OR, 0.92; 95%CI, 0.32–2.63) and adjusted analysis (Adjusted OR, 1.32; 95%CI, 0.41–4.22). For cure rate, there was a statistically significant effect before (OR, 93.34; 95%CI, 29.53–295.99) and after adjusted analysis (Adjusted OR, 86.24; 95%CI, 27.05–274.94), although imprecise. In PSM analysis, SSM completion was associated with increased odds of cure (OR, 87.00; 95%CI, 12.12–624.59) but not TSR (OR, 1.67; 95%CI, 0.40–6.97).

**Conclusions:**

Completing SSM increases cure but has no effect on TSR among adult BC-PTB patients in eastern Uganda. Implementation of SSM should be encouraged to ensure improvement in cure rates among tuberculosis patients in rural areas.

## Introduction

Tuberculosis (TB) is among the top 10 causes of death globally,[1] and among people living with Human Immunodeficiency Virus (HIV), it is ranked the number one cause of death.[1, 2] The most recent World Health Organization (WHO) Global TB report released 2019 indicates that 10 million people developed TB disease in 2018,[1] of which one and half million died.[1] The highest burden of TB is in sub Saharan Africa (SSA), a region with the slowest decline in TB incidence rate.[3] Moreover, TB is curable and successfully treatable with standardized short course treatment. Good TB program performance results into high treatment success rate (TSR) matching the WHO target of at least 90% TSR and 85% cure rate.[3] Achieving a high TSR is a challenge across several TB programs globally. For instance, the global TSR for new bacteriologically confirmed pulmonary TB (BC-PTB) patients fell from 83% in 2015 to 82% in 2016 according to the 2018 Global TB report.[3] However, the 2019 Global TB Report indicates TSR improved to 85%,[1] still lower than the optimum target of at least 90%.

Standard 10 of the International Standards of TB care requires that all BC-PTB patients who are started on anti-TB medications should receive treatment monitoring with follow-up sputum smear microscopy tests at two, five, and six months.[4] This sputum smear monitoring (SSM) is critical in establishing treatment response, detecting early treatment failures, and drug resistances among TB patients. Presently, the gold standard test for assessing treatment response is sputum smear microscopy since culture and GeneXpert tests have not been validated for the same purpose.

Uganda is one of the 30 high TB-HIV burdened countries as per the WHO classification because it contributes at least 1,000 incident TB-HIV cases per year,[5] and has suboptimal TB treatment outcomes as well. Recent data indicates a TSR of 80% and cure rate of 48%,[6] which are far below the WHO desired targets. In addition, there are substantial differences in TSR across districts and health facilities. For instance, in rural eastern Uganda, a TSR of less than 80% was reported in Soroti and Kumi districts while a TSR of more than 90% was reported in Serere and Ngora districts.[6] Accordingly, Uganda’s Ministry of Health suggested that incomplete SSM and health systems challenges contributes to the observed suboptimal rates of cure and treatment success.[7] However, this hypothesis is supported by empiric data, although treatment monitoring is critical for improved cure rates among TB patients.[8] It is also unclear whether completion of SSM has effect on TSR, and whether it can explain the observed differences.

In previous observational studies conducted in Uganda [9] and Ethiopia [10], data showed completion of SSM has an effect on TSR. However, the application and generalizability of these results are limiting because of imbalanced participant characteristics between completers and non-completers of SSM due to lack of randomization, leading to biased estimates in treatment effects. Second, the inherent limitations of observational studies namely selection biases and confounding raises concerns regarding the validity of these results. One approach to achieve balance in participant characteristics, reduce selection bias and confounding, and to estimate a less-biased outcome is to use propensity score matched (PSM) analysis. [11, 12]

The purpose of the present study was therefore to evaluate the effect of completion of SSM on TSR among adult BC-PTB patients in rural eastern Uganda via PSM analysis. We hypothesized that there is a difference in TSR between adult BC-PTB patients who complete SSM and those who do not. Our study will provide evidence for designing interventions that addresses suboptimal differences in TSR among adult BC-PTB patients, between districts and health facilities in Uganda, and similar settings in sub Saharan Africa.

## Methods and materials

### Study reporting

We followed the guidelines on analysis and reporting of PSM analysis [13] in the design, conduct, and analysis of this study. This study presents a secondary data analysis of a retrospective cohort study conducted in Eastern Uganda. We received a waiver of informed consent to access the TB patient records as the number of patients was very large and could not be reached individually.

### Description of data source

We constructed a retrospective cohort study using routinely collected medical records. The study participants were adult (15 years and over) BC-PTB patients, both new and retreatment cases who were diagnosed and treated between January 2015 and June 2018 across 10 health facilities in rural Eastern Uganda. Participants received TB care according to the Uganda National TB and Leprosy Treatment Guidelines.[14] Data were abstracted on patient clinical characteristics that health workers record in the TB unit register, both for new and retreatment BC-PTB patients (S1 File).

The variables adopted for analysis included: grouped age (15 to 24, 25 to 34, 35 to 44, 45 to 54, and 55+ years), sex (male or female), anti-TB regimen (category I or category II), pre-treatment bacilli load (≤2+, 3+, and GeneXpert status), form of Directly Observed Therapy Short Course (DOTS) whether health facility or community, type of TB patient (new or retreatment case), and whether the participant had a treatment supporter or not. Treatment supporters are trusted and respected persons selected among members of the community (family member or friend) with whom a TB patient declares good mutual relationship. Their role is to support TB patients in treatment adherence and completion, and sputum smear follow-up testing among others.[7] Additional variables adopted included SSM (complete or incomplete), health facility location (urban or rural), health facility ownership (public or private not for profit), and the level of TSR in the district whether low (below 90%) or high (above 90%). We used a threshold of 90% because WHO required a TSR of 90% and beyond as a benchmark for good TB program performance.

### Study design and rationale

We used PSM analysis to create a quasi-experimental study from the observational data. Propensity score is the probability of receiving an intervention by participants in the untreated group, participants with incomplete SSM in our study, based on observed set of participant characteristics.[15] The scores range from zero to one.[16] This statistical approach allowed us to balance the two non-equivalent groups, incomplete and complete SSM groups, on observed participants characteristics, except for the treatment.[17] PSM analysis does not create a true randomization process but aims to replicate one, hence a quasi-experimental study design.[17] Use of PSM has important implications. First, PSM analysis reduces treatment selection bias resulting from participant characteristics and residual confounding, which are major limitations to establishing treatment effects in observational epidemiological studies. Second, in observational studies, the treatment and control groups differ systematically on observed participant characteristics due to lack of randomization at assignment into the treatment groups leading to a biased estimate of the treatment and outcome relationship.[18] PSM analysis mitigates this problem by simulating a randomization of participants to treatment groups using propensity scores, reducing selection bias and confounding, and estimating a less-biased treatment effect(s).[19–21]

#### Exclusion criteria

The study excluded patients with clinically diagnosed or extra pulmonary TB because their response to treatment was monitored using clinical assessments such as improvement in nutritional status, resolution of TB symptoms among others, but not SSM tests. We also excluded adult BC-PTB patients who were transferred-in after the intensive phase of TB treatment (2 months for new BC-PTB patients and 3-months for retreatment BC-PTB patients) because it was difficult to ascertain their SSM status at the referring health facility.

### Measurements

#### Outcome variables

Our primary outcome was TSR among adult BC-PTB patients, defined as adult BC-PTB patients who were either cured or who had completed TB treatment. The secondary outcome was cure rate, defined as having a negative SSM test result at the end of TB treatment, and on one (either at five or two months) or more previous occasions (both five and two months).

#### Treatment variable

The treatment variable or intervention was completion of SSM. Participants who received all the three SSM tests in the course of TB treatment at two, five, and six months for new BC-PTB patients, or at three, five, and eight months for retreatment BC-PTB patients were considered to have had complete SSM, otherwise incomplete SSM. Accordingly, this study referred to these participants as “Complete SSM group” and “Incomplete SSM group”, respectively.

#### Covariates (matching variables)

We selected 10 covariates for estimating propensity scores. These covariates were biologically or empirically related to the outcome,[22] an approach that ensures assignment to treatment is independent of the outcome conditional on the covariates.[23] The 10 covariates included age group, sex, anti-TB regimen, pre-treatment bacilli load, form of DOTS, type of TB patient, TB treatment supporter, health facility location, health facility ownership, and level of TSR in the district.

### Statistical analysis

#### Baseline comparison of participant characteristics

The statistical analysis was performed in Stata version 15 (StataCorp, TX, USA).[24] We summarized categorical data into frequencies and percentages, and numerical data into means and standard deviations. Differences in the distribution of observed participant characteristics between the complete and incomplete SSM groups in the unmatched and matched samples were compared using standardized mean differences (SMD). We considered SMD greater than 0.1 as indicative of statistically significant difference, and vice-versa. We did not use Chi-square or Fisher’s exact, Mann-Whitney U or the Student’s t-tests to compare differences in baseline characteristic because the associated p-values are sample size dependent. Accordingly, these tests are not recommended for checking covariate balance.[25]

#### Estimation of propensity scores

We estimated propensity scores in a logit model by regressing the treatment, i.e. completion of SSM, on the matching covariates. The propensity scores were then the predicted probabilities of treatment derived from fitting the logit model.[26] We assessed initial balance in propensity scores and covariates between the complete and incomplete SSM groups by splitting the sample into equally spaced intervals. Within each interval, the Student’s t-test was used to evaluate statistically significant differences in the average propensity scores between the groups. When statistically significant differences were observed, the intervals were divided further and re-tested until no differences in propensity scores were present. We checked the degree of overlap of propensity scores and covariates between the complete and incomplete SSM groups using propensity score graph. We achieved covariate balance, and therefore did not seek higher order or interaction terms.[23]

#### Propensity score matching and covariate balance

We matched participants in the complete SSM group to those in the incomplete SSM group on similar propensity scores using several approached namely: one to one (pair) matching with and without replacement, nearest neighbor 1:1 caliper, radius, and kernel matching. In one to one (nearest neighbor) PSM, completers of SSM were matched to non-completers regardless of how poor the match was. In caliper PSM, completers of SSM were matched to non-completers within a caliper of 20% of the standard deviation of the propensity score in 1:1 ratio to prevent bias from distant matches.

In radius PSM, the matching was performed not merely on the nearest neighbor within the defined caliper but on all participants within the caliper, thus allowing the use of several available matches. In Kernel matching/or weighting, the weighted average of all participants in the incomplete SSM group was used to construct the missing counterfactual outcome to enable the use of more data and produce less differences. However, this comes along with poor matches as a pitfall.[27]

Although we explored several approaches to matching, the most appropriate matching approach selected was based on the following criteria: 1) balance of all covariates across the complete and incomplete SSM groups; 2) significant reduction in propensity score pseudo R-square value; 3) reduction in mean standardized difference in covariate between the complete and incomplete SSM groups to less than 5%.[25] After matching, we again checked covariate balance across the complete and incomplete SSM groups with standardized mean differences (SMD), and considered SMD less than 0.1 as indicative of good covariate balance.[28]

#### Outcome analysis

We estimated the effect of completion of SSM on TSR in the unmatched sample with binary logistic regression analysis, without and with adjustment for potential confounders (matching covariates). We reported the results as crude and adjusted odds ratios, respectively. In the matched sample, we used conditional logistic regression analysis to determine the effect of completion of SSM on TSR, and considered matched pairs in the analysis. We reported the results as odds ratio with corresponding 95% confidence intervals (CIs). All outcome analyses were performed at 5% statistical significance level.

#### Sensitivity analysis

Since PSM relies on conditional independence or unconfoundedness assumption, we checked the sensitivity of the results with respect to deviations from this assumption. We performed Mantel-Haenszel (MH) bounding approach proposed by Rosenbaum using Stata command “*mhbound*” to determine the strength of the influence of unmeasured variables on the selection process, and to check the sensitivity of the estimated average treatment effects in the treated group.[29]

#### Human subjects’ issues

This study was reviewed and received approval from Mbarara University of Science and Technology Research Ethics Committee (Reference number 03/11-18) and the Uganda National Council for Science and Technology (Reference number HS 2531). Final permission to collect data was granted from the Office of the President (Reference number ADM 194/212/01). The need for patient consent was waived by the Ethics Committee because the records to be reviewed were numerous and it was not logistically possible to reach all the participants.

## Results

### Study profile and participant characteristics before propensity score matching

**Fig 1:**
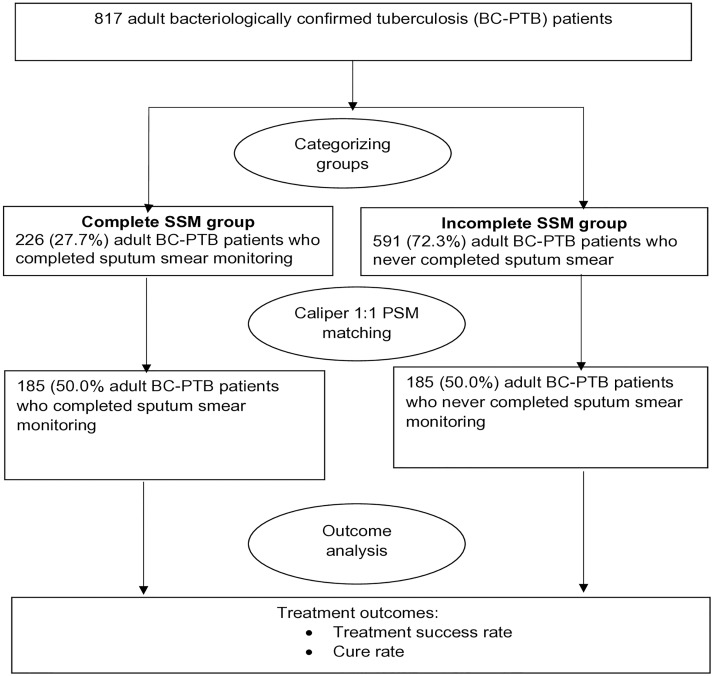
Study profile showing the number of adult bacteriologically confirmed tuberculosis cases before and after propensity score matching, rural Eastern Uganda.

Overall, we abstracted records for 817 patients. In this unmatched sample (Fig 1), 591 (72.3%) participants were in the incomplete SSM group while 226 (27.7%) were in the complete SSM group.

Table 1 shows the distribution of participant characteristics before and after PSM. Before PSM, several participants’ characteristics varied considerably between the complete and incomplete SSM groups with SMD exceeding the 0.1 limit. The complete and incomplete SSM groups were only similar with respect to sex (SMD = 0.087) and anti-TB regime (SMD = 0.005) since the SMD was less than 0.1. However, after matching participants in the incomplete SSM group to those in the complete SSM group on similar propensity scores, all the characteristics became similar between participants in the complete and incomplete SSM groups (all SMD was less than 0.1). Thus 185 (50.0%) participants in the complete SSM group were matched to 185 (50.0%) participants in the incomplete SSM group on similar propensity scores.

**Table 1:** Patient characteristics before and after propensity score matching.

		Before propensity score matching			After propensity score matching		
Variables	Level	Incomplete SSM n = 591 (%)	Complete SSM n = 226 (%)	SMD	Incomplete SSM n = 185 (%)	Complete SSM n = 185 (%)	SMD
Health facility ownership	Public	509 (86.1)	214 (94.7)	0.294	177 (95.7)	175 (94.6)	0.05
	PNFP	82 (13.9)	12 (5.3)		8 (4.3)	10 (5.4)	
Health facility location	Rural	202 (34.2)	29 (12.8)	0.52	23 (12.4)	26 (14.1)	0.048
	Urban	389 (65.8)	197 (87.2)		162 (87.6)	159 (85.9)	
Level of TSR in the district	Low	364 (61.6)	190 (84.1)	0.522	153 (82.7)	153 (82.7)	<0.001
	High	227 (38.4)	36 (15.9)		32 (17.3)	32 (17.3)	
Sex	Male	373 (63.1)	152 (67.3)	0.087	118 (63.8)	122 (65.9)	0.045
	Female	218 (36.9)	74 (32.7)		67 (36.2)	63 (34.1)	
Age group (years)	15–24	115 (19.5)	54 (23.9)	0.163	38 (20.5)	39 (21.1)	0.044
	25–34	155 (26.2)	64 (28.3)		61 (33.0)	58 (31.4)	
	35–44	125 (21.2)	47 (20.8)		36 (19.5)	38 (20.5)	
	45–54	103 (17.4)	29 (12.8)		25 (13.5)	26 (14.1)	
	55+	93 (15.7)	32 (14.2)		25 (13.5)	24 (13.0)	
Age (mean (SD))		38.66 (15.29)	36.81 (14.99)	0.122	37.05 (15.29)	36.71 (14.53)	0.023
Type of TB patient	New	528 (89.3)	194 (85.8)	0.106	173 (93.5)	172 (93.0)	0.022
	Retreatment	63 (10.7)	32 (14.2)		12 (6.5)	13 (7.0)	
Pre-treatment TB bacilli load	≤2+	251 (42.5)	84 (37.2)	0.252	79 (42.7)	74 (40.0)	0.059
	+3	198 (33.5)	62 (27.4)		53 (28.6)	54 (29.2)	
	GeneXpert	142 (24.0)	80 (35.4)		53 (28.6)	57 (30.8)	
Anti-TB regimen	Category I	549 (92.9)	213 (94.2)	0.055	179 (96.8)	178 (96.2)	0.029
	Category II	42 (7.1)	13 (5.8)		6 (3.2)	7 (3.8)	
Type of DOTS	Facility	30 (5.1)	4 (1.8)	0.183	2 (1.1)	1 (0.5)	0.06
	Community	561 (94.9)	222 (98.2)		183 (98.9)	184 (99.5)	
TB Treatment Supporter	Yes	499 (84.4)	218 (96.5)	0.418	181 (97.8)	180 (97.3)	0.035
	No	92 (15.6)	8 (3.5)		4 (2.2)	5 (2.7)	

Note: Category I: 2RHZE/6HE and 2RHZE/4RH; Category II: 2RHZES/1RHZE/5RHE; SMD: Standardized Mean difference

### Selection of matching method and balance diagnostics

Table 2 shows among several matching techniques used, the nearest neighbor 1:1 caliper PSM was the best since all the covariates were balanced across the complete and incomplete SSM groups, the PSM pseudo R-squared value reduced significantly from 0.104 to 0.004, and percentage bias dropped from 20 to 2.6. Also, the standardized percentage bias across covariates before and after matching was less than 5%, indicating good covariate balance. The rest of the matching techniques did not achieve good balance of all covariates between the complete and incomplete SSM groups.

**Table 2:** Sample sizes, means, standardized differences across all covariates before and after matching, and rationale for choice of matching method.

Sample type	Sample size	Number of participants who completed SSM	Number of participants who never completed SSM	Mean Standardized Difference in covariates	Median Standardized difference in covariates	Pseudo R-Square value	Rationale
Original (unmatched) sample	817	226	591	20.0	13.0	0.104	Several covariates were not balanced between the complete and incomplete SSM groups.
One to one matched sample, with no replacement	452	226	226	3.3	1.7	0.009	Three covariates were not balanced complete and incomplete SSM groups
One to one matched sample, with replacement	64	32	32	4.3	2.9	0.013	Three covariates were not balanced between the complete and incomplete SSM groups, and a large drop in sample size occurred.
Radius and caliper matching	64	32	32	5.4	3.9	0.015	Three covariates were not balanced between the complete and incomplete SSM groups. Larger drop in sample size.
Kernel matched sample	817	226	591	1.4	0.9	0.002	Retained all observations but had poor balance of covariates between the complete and incomplete SSM groups
Nearest neighbor caliper matched sample	370	185	185	2.6	2.4	0.004	All covariates were balanced between the complete and incomplete SSM groups.

### Propensity score estimation results

Table 3 shows results for completion of SSM regressed on TSR in a logit model. The results showed pre-treatment bacilli load, TB treatment support, location of health facility, and the district TSR rating were significantly associated with selection of completion of SSM (all *p*<0.05).

**Table 3:** Propensity score estimation results in a logit model.

Variables	Level	Coefficient	95% CI for coefficient
Health facility (Ref: Public)	Private Not for Profit	-0.22	(-1.07,0.63)
Sex (Ref: Male	Female	-0.28	(-0.63,0.08)
Age group in years (Ref: 15–24 )	25–34	-0.16	(-0.62,0.31)
	35–44	-0.29	(-0.79,0.21)
	45–54	-0.45	(-1.01,0.11)
	≥55	-0.09	(-0.65,0.47)
Anti-TB regimen (Ref: Category I)	Category II	-0.80	(-1.74,0.14)
Type of TB patient (Ref: New BC-PTB)	Retreatment BC-PTB	0.50	(-0.20,1.21)
MTB load (Ref: ≤2+)	3+	0.10	(-0.30,0.50)
	By GeneXpert	0.66**	(0.24,1.07)
Type of DOTS (Ref: Facility)	Community	0.61	(-0.53,1.75)
Treatment supporter (Ref: Yes)	No	-1.23**	(-2.01,-0.46)
Health facility site (Ref: Rural)	Urban	0.67*	(0.05,1.29)
Level of TSR in the district (Ref: Low)	High	-1.02***	(-1.51,-0.53)
Number of observations		817	

Note: 95% confidence intervals in brackets; Category I: 2RHZE/4RH and 2RHZE/6HE; Category II: 2RHZES/1RHZE/6RHE; DOTS: Directly Observed Therapy Short Course; MTB: Mycobacterium tuberculosis;

*p* < 0.05,

** *p* < 0.01,

*** *p* < 0.001 at 5% significance level;

Ref: Reference category; TSR: Treatment success rate.

### Effect of SSM completion on TSR

Table 4 shows the results for the effect of completion of SSM on TSR. In the unmatched sample, completion of SSM was associated with a statistically non-significant reduction in TSR at unadjusted analysis (OR, 0.92; 95% CI, 0.32–2.63) and a statistically non-significant increase in TSR after adjusting for potential confounders (Adjusted OR, 1.32; 95% CI, 0.41–4.22). In the propensity score matched sample, there was no statistically significant increase in TSR as well (OR, 1.67; 95% CI, 0.40–6.97).

**Table 4:** Matched and unmatched analysis for effect of SSM on TSR.

Outcome analysis	TSR (OR, 95% CI		Cure (OR, 95% CI)	
Unadjusted unmatched	0.92	(0.32,2.63)	93.34***	(29.54,294.99)
Adjusted unmatched	1.32	(0.41,4.22)	86.24***	(27.05,274.94)
PS matched	1.67	(0.40,6.97)	87.00***	(12.12,624.60)

Note: 95% confidence intervals in brackets;

*** *p* < 0.001 at 5% significance level

In sub-group analysis (Table 4), we examined the effect of completion of SSM on cure rate. Our analysis indicated a statistically significant effect of completion of SSM on cure rate at unadjusted (OR, 93.34; 95% CI, 29.53–294.99), adjusted (adjusted OR, 86.24; 95% CI, 27.05–274.94), and PSM analyses (OR, 87.00; 95% CI, 12.12–624.59).

### Sensitivity analysis results

Sensitivity analysis showed our results were robust to hidden bias since a large increase of 1.5 in the lower bounds of odds of differential assignment due to unobserved factors (Gamma) was needed for the result to shift from a statistically non-significant (Gamma = 1, *p* = 0.360) to statistically significant result (Gamma = 2.5, *p* = 0.043). A large change in Gamma that causes a shift from non-significant to significant result is a confirmation of robust finding to hidden bias.[30]

## Discussion

Our study examined the effect of completing sputum smear monitoring on rates of treatment success and cure among adult BC-PTB patients in rural eastern Uganda, and the data showed that completion of SSM was not associated with TSR but with increased odds of cure. Studies to examine the same research question have showed different results. One epidemiological study conducted in Kiboga and Kyankwazi districts in Uganda showed BC-PTB patients who never completed SSM had increased odds of unsuccessful TB treatment compared to those who completed.[9] In Ethiopia, TB patients who received sputum smear follow-up tests were more likely to achieve treatment success compared to those who had not.[10] In India, nearly 62% of successfully treated pulmonary TB patients completed SSM.[31]

Our results are different for several reasons. First, the observational studies in Uganda and Ethiopia [9,10] had inherent study design limitations such as selection bias, confounding, and residual confounding.[32] The studies did not balance observed covariates between the complete and incomplete SSM groups in establishing unbiased treatment effects, as recommended.[29] In contrast, our study used PSM analysis approach to obtain a less-biased estimate of the effect of completing SSM on TSR. Second, with respect to the study in India,[31] the conclusion of an association between SSM and TSR was based on descriptive analysis which is potentially biased. Third, the results of the Indian study should be cautiously compared to ours because it did not control for potential confounders at analysis.

Our results are comparable to those in a study in Malawi which also did not demonstrate an association between SSM completion and TSR. However, the Malawian study had imprecise time points for establishing the status of SSM.[33] The time points for performance of SSM were as follows: two or three months for two months SSM; four, five or six months for five months SSM; and six, seven, eight or nine months for six or eight months SSM.[33] Clearly, the Malawian study had less-stringent and imprecise time points for assessing SSM compared to the present study, and the possibility of bias in the results cannot be ignored.

### Implications of study findings

Our findings have important implications for TB clinical practice, research, monitoring and evaluation (M&E), and programming in areas similar to eastern Uganda from where these data were collected. Our data did not demonstrate an effect of inadequate completion of SSM on TSR. Instead, it showed that incomplete SSM impacts cure suggesting TB control programs should hence focus on enhancing treatment adherence in order to achieve better sputum smear conversion rates and eventually high cure rates. In clinical practice, healthcare providers should emphasize the importance of SSM to patients as completion is associated with increased odds of cure and subsequently good TB program performance. Since our study showed no effect of completion of SSM on TSR but an effect on cure rate, large and robust epidemiological studies perhaps should be conducted to approve or disapprove these effects. In TB programs, an M&E indicator on completion of SSM should be added to existing ones to enable practitioners, researchers, policy makers, and health managers in tracking treatment monitoring among TB patients.

### Study strengths and limitations

Our study used a robust methodological approach to estimate unbiased effect of completing SSM on TSR and cure rate. However, the interpretation of these findings requires a number of considerations. First, our study was able to balance observed but not unobserved covariates between completers and non-completers of SSM. The results thus hold true for only observed participant variables although our sensitivity analysis indicated these findings are robust to hidden bias. Ideally, an RCT would have been the best design to achieve the study objective but is not feasible for ethical reasons. Second, the analytic approach reduced the sample size and this potentially limits the generalizability of findings because a larger sample size is preferred. Third, reduced sample sizes are known to increases the risk of type II error.[34] Nevertheless, the remaining sample size met the commonly recommended minimum of 10(p+1), where p is the number of matching variables.[35,36] Lastly, the validity of sputum smear microscopy tests is a concern since this study could not validly conclude on the quality of sputum smear examinations with respect to false negative and false positive results. This concern is not unique to this study, but was highlighted in a previous study in India.[37] However, our data are from health facilities participating in internal and external quality assurance activities of the National TB and Leprosy Control Program.

Despite these limitations, data in the present study demonstrated the importance of completing SSM on rates of treatment success and cure among adult BC-PTB patients, and the application of PSM in observational studies in TB programs. The study has generated sound scientific evidence for designing prospective research studies, formulating policy, and improving clinical practice and TB programming.

## Conclusions and recommendations

Our study shows that completing SSM is associated with increased odds of cure rate but has no effect on TSR among adult BC-PTB patients in rural eastern Uganda. We recommend that completion of SSM among adult BC-PTB patients should be enforced by TB programs for improved cure rates among adult BC-PTB tuberculosis patients in rural areas of Uganda and similar settings in sub Saharan Africa.

## Supporting information

S1 File Dataset. (DTA)
